# Shared functional network abnormality in patients with temporal lobe epilepsy and their siblings

**DOI:** 10.1111/cns.14087

**Published:** 2023-01-17

**Authors:** Kangrun Wang, Fangfang Xie, Chaorong Liu, Ge Wang, Min Zhang, Jialinzi He, Langzi Tan, Haiyun Tang, Fenghua Chen, Bo Xiao, Yanmin Song, Lili Long

**Affiliations:** ^1^ Department of Neurology, Xiangya Hospital Central South University Changsha China; ^2^ Department of Neurosurgery, Xiangya Hospital Central South University Changsha China; ^3^ Clinical Research Center for Epileptic disease of Hunan Province, Xiangya Hospital Central South University Changsha China; ^4^ National Clinical Research Center for Geriatric Disorders, Xiangya Hospital Central South University Changsha China; ^5^ Department of Radiology, Xiangya Hospital Central South University Changsha China; ^6^ Department of Emergency, Xiangya Hospital Central South University Changsha China

**Keywords:** endophenotype, functional magnetic resonance imaging, graph theory, independent component analysis, temporal lobe epilepsy

## Abstract

**Aim:**

Temporal lobe epilepsy is a neurological network disease in which genetics played a greater role than previously appreciated. This study aimed to explore shared functional network abnormalities in patients with sporadic temporal lobe epilepsy and their unaffected siblings.

**Methods:**

Fifty‐eight patients with sporadic temporal lobe epilepsy, 13 unaffected siblings, and 30 healthy controls participated in this cross‐sectional study. We examined the task‐based whole‐brain functional network topology and the effective functional connectivity between networks identified by group‐independent component analysis.

**Results:**

We observed increased global efficiency, decreased clustering coefficiency, and decreased small‐worldness in patients and siblings (*p* < 0.05, false discovery rate‐corrected). The effective network connectivity from the ventral attention network to the limbic system was impaired (*p* < 0.001, false discovery rate‐corrected). These features had higher prevalence in unaffected siblings than in normal population and was not correlated with disease burden. In addition, topological abnormalities had a high intraclass correlation between patients and their siblings.

**Conclusion:**

Patients with temporal lobe epilepsy and their unaffected siblings showed shared topological functional disturbance and the effective functional network connectivity impairment. These abnormalities may contribute to the pathogenesis that promotes the susceptibility of seizures and language decline in temporal lobe epilepsy.

## Graphical abstract

Patients with temporal lobe epilepsy and their unaffected siblings showed shared topological disturbance of whole‐brain functional network and the effective functional network connectivity impairment between the ventral attention network and the limbic system. These abnormalities were potential endophenotype candidates and may contribute to the pathogenesis that promotes the susceptibility of seizures and language decline in temporal lobe epilepsy.

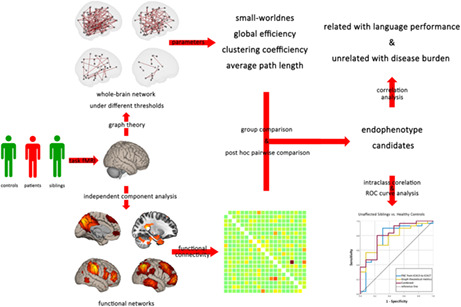

## 1 INTRODUCTION

Temporal lobe epilepsy (TLE) is the most common drug‐resistant focal epilepsy syndrome in adults,^1^ pathologically characterized by hippocampal sclerosis (HS) in two‐thirds of the cases.^2^ Previous MRI studies proposed the idea that TLE was a network disorder. Specifically, TLE was related to atrophy of neocortex,^3^, ^4^, ^5^ cerebellar,^6^ hippocampus^5^ and thalamus,^7^, ^8^ and derangements of structural^7^, ^9^ and functional connectivity^8^, ^10^ across the whole brain. It remains unresolved whether the network disruptions are consequences or predisposing risk factors for continued seizures.

Endophenotypes are heritable, measurable characteristics that link genotype to phenotype.^5^, ^11^ Studying endophenotypes help deconstruct complex clinical symptoms and increase the analytical power of genetic mapping for polygenetic disorders such as sporadic TLE. Since endophenotypes have a higher prevalence in unaffected family members of patients than in the normal population, studying unaffected siblings of patients is a popular approach to explore endophenotypes.^5^, ^9^, ^12^, ^13^ In our previous study, we observed shared hippocampal distortion in patients with TLE and their unaffected siblings.^5^ Other studies also identified white matter and thalamic atrophy,^14^ temporal morphological alteration,^15^, ^16^ and decreased mean diffusivity in left superior longitudinal fasciculi and corticospinal tract^9^ in the affected/unaffected sibling pairs of TLE. However, how do these structural endophenotypes lead to functional malfunction, including seizure propagation and language decline, is unresolved.^17^ Functional endophenotypes are yet to be explored to link structural endophenotypes to clinical symptoms.^18^, ^19^

Graph theory provided insights into the topological disturbance in TLE and mathematical tools to quantitatively characterize comprehensive cerebral networks. Functional brain network topology modulated the threshold for seizure propagation,^20^ surgical outcomes,^6^, ^21^, ^22^ and cognitive function^23^ in patients with TLE. Previous graph theoretical works on cortical thickness,^6^, ^22^ white matter tracts,^22^, ^24^ and functional MRI^21^, ^23^, ^25^, ^26^ advanced the knowledge of TLE as a network disorder, showing a less small‐world topology in TLE. While graph theory unfolds the functional network at the whole‐brain level, group‐independent component analysis (group‐ICA) separates the whole brain into functional segregated networks.^27^, ^28^ Then, effective functional network connectivity (eFNC) could probe into directed connectivity from one network to another network after discounting the effect by other networks. Abnormal connections between limbic system, default mode network (DMN), and attention networks contributed to the comorbidity of cognitive decline.^10^, ^29^

While resting‐state functional MRI focuses primarily on the spontaneous activities in rest, task‐based functional MRI (tb‐fMRI) provides direct information for language impairment.^30^ Notably, verbal fluency tasks mobilize a network roughly overlapping with the hippocampal epileptogenic network, providing an ideal tool to study the underlying malfunctional network and explore potential functional endophenotypes in TLE.^8^, ^31^

With a well‐tested Chinese character verbal fluency task,^31^, ^32^ this study aimed to explore the shared task‐related functional network disorders in patients with TLE and their unaffected siblings. We applied graph theoretical approaches to investigate the topology of the whole‐brain functional network. We analyzed the effective connectivity between networks identified by group‐ICA. At last, we used three approaches to test their potential as functional endophenotypes.

## 2 MATERIAL AND METHODS

### 2.1 Subjects

From December 2018 to January 2021, we sequentially recruited 58 patients with sporadic TLE from the Department of Neurology, Xiangya Hospital. Patients visited our institution due to recent seizures. We identified 30 patients with HS (TLE‐HS, left TLE (LTLE):right TLE (RTLE) = 13:17) and 28 patients without HS (TLE‐NHS, LTLE:RTLE = 12:16). The clinical semiology, electroencephalography, and MRI evidence were evaluated by two experienced epileptologists (BX and LL) to diagnose and lateralize TLE.^33^ Thirteen^5^, ^12^ unaffected siblings of patients participated in this study. Thirty age, sex, and educational level matched healthy controls (HC) were recruited from the same social background. For all subjects, the exclusive criteria included (1) brain lesions other than HS; (2) history of neurological or psychiatric disease except for TLE; (3) under 16 or over 65 years of age; (4) unable to comprehend our language paradigm. All subjects are right‐handed native Chinese speakers.

All subjects routinely underwent T2WI, T2WI fluid‐attenuated inversion recovery, and 3DT1 sequences, which were visually assessed by experienced neuroimagers to diagnose HS or other potential structural abnormalities. The hippocampi of subjects were segmented by Hipposeg, an automatic online hippocampal segmentation algorithm developed for TLE, to calculate the hippocampal volume (http://niftyweb.cs.ucl.ac.uk/program.php?p=HIPPOSEG),^34^ which was then corrected for total intracranial volume. The 95% confidence interval (CI) of HC was calculated as the reference volume. Hippocampal sclerosis was defined as (1) visually decreased hippocampal volume, asymmetrical hippocampus, or loss of internal structure on T1‐weighted MRI; (2) increased signal in hippocampi on T2‐weighted MRI; and (3) smaller than the reference hippocampus volume. Criteria one and two were carried out by two neuroimager specialized in TLE (FF and HY). According to a well‐established protocol,^35^ disagreement between the neuroimagers and automatic segmentation was reconciled by a blind rater (LL) who has rich experience in both visual assessment and automatic segmentation.^5^

Written informed consent was obtained from all participants. The study was approved by the ethics committee of Xiangya Hospital of Central South University.

### 2.2 Neuropsychological tests

Participants routinely underwent neuropsychological tests, including the Self‐Rating Anxiety Scale (SAS),^36^ Self‐Rating Depression Scale (SDS),^37^ Boston Naming Test (BN), Montreal Cognitive Assessment (MoCA),^38^ and verbal fluency Pinyin test (VFP).^32^ Before the tb‐fMRI scan, subjects were given a verbal fluency Character test (VFC),^32^ same as our language task, to confirm that they understand the procedure and estimate their performance during the scan.

### 2.3 MR data acquisition

With a Siemens MAGNETOM Prisma 3.0T MR scanner and standard head coils at the MRI center of Xiangya Hospital, MRI images were collected, including the whole‐brain structural images obtained using a magnetization‐prepared rapid acquisition with gradient echo sequence (field of view 233 mm, repetition time [TR] = 2.11 s, echo time [TE] = 3.18 ms, flip angle = 9°, 320 × 320 matrix), and the whole‐brain blood oxygenation level‐dependent (BOLD) signals provided by a gradient echo planar T2‐weighted sequence (field of view 225 mm, TR = 1 s, TE = 37 ms, flip angle = 52°, 90 × 90 matrix).

A previously described and well‐tested Chinese character verbal fluency task^31^, ^32^ was carried out during the fMRI scan. Subjects needed to silently come up with words that initiate with the single Chinese character exhibited on the screen.

### 2.4 fMRI image preprocessing

Imaging data were preprocessed with the Statistical Parametric Mapping 12 software (https://www.fil.ion.ucl.ac.uk/spm/). The preprocessing pipeline includes realignment, coregistration, segmentation, normalization to the Montreal Neurological Institute (MNI) space, and spatial smoothing (6 mm).^31^

### 2.5 Whole‐brain functional connectivity and graph theory analysis

We used toolbox CONN v.20.b^39^ (http://www.nitrc.org/projects/conn) to conduct two different types of functional connectivity analysis with our tb‐fMRI data. Task‐based functional connectivity (tb‐FC) analyzed the functional connections during tasks. By regressing out task‐specific signals, the task residual‐functional connectivity (tr‐FC) approach extracted resting‐state functional connectivity from tb‐fMRI data.^31^

The image data of every subject were parcellated into 272 regions of interest (ROI), including 210 cortical region, 36 subcortical regions,^40^ and 26 cerebellar regions.^41^ Outlying scans were detected by the functional outlier detection tool embedded in the CONN. Head motion, outlying scans, the effect of modules, and BOLD signals inside white matter and cerebral spinal fluid were defined as confounders and were regressed out.^39^ BOLD signals were filtered with a band‐pass filter [0.009–0.08] Hz to remove task‐evoked signals in the tr‐FC analysis.^31^ A wider [0.009–0.10] Hz band‐pass filter was applied for tb‐FC analysis.^42^

A generalized linear model (GLM) with bivariate‐correlation^39^ was used for the individual‐level analysis. The Pearson correlation coefficients were calculated for every pair of ROIs to generate a 272 × 272 weighted matrix for each subject. Weighted matrices were processed into binarized undirected matrices with connection density ranging from 5% to 40%, in steps of 1%.^8^ For each connection density, we explored four global metrics: (1) global efficiency (GE); (2) average shortest path length (APL); (3) clustering coefficiency (CC); (4) small‐worldness (σ) = (CC/CC_rand_)/(APL/APL_rand_). Both GE and APL reflect the integration level of the network, and the CC measures the segregation level of the network. The property of healthy brains accords with a small‐world organization, characterized by relatively higher CC and lower APL than that of a random network. CC_rand_ and APL_rand_ are the CC and APL of equivalent random networks. The theoretical values are:

$$\begin{array}{c} \begin{array}{c} {\mathrm{CC}}_{\mathrm{rand}}=\frac{\text{average degrees of nodes}}{\text{number of nodes}};{\mathrm{APL}}_{\mathrm{rand}}=\frac{\ln\left(\text{number of nodes}\right)}{\ln\left(\text{average degrees of nodes}\right)}. \end{array} \end{array}$$

To investigate the between‐group differences across different connection densities, we computed the area under curve (AUC), which provided an overall scalar value integrating differences under all densities.

### 2.6 Group‐ICA and post hoc eFNC

The toolbox CONN v.20.b was used to conduct group‐ICA and post hoc eFNC.^27^, ^39^ According to previous studies, the whole brain was divided into 20 independent components (ICs) to identify the DMN and task‐related networks.^27,28,43^ ICs were labeled by the dice coefficient of spatial overlap and visual assessment.^28^, ^43^ Then, group‐level ICs were reconstructed into individual‐level ICs.^27^ The time courses in individual‐level ICs were extracted for eFNC analysis. The denoising and filtering steps were the same as tb‐FC described above. GLM with semipartial‐correlation was used to construct individual‐level eFNC matrices.^39^ Effective FNC was compared between three groups by analysis of covariance (ANCOVA) and post hoc pairwise comparisons, with age, sex, educational level, and MoCA controlled as covariates of no interest. Connectivity was considered significant under a threshold of false discovery rate (FDR)‐corrected, *p* < 0.05.

### 2.7 Statistical analysis

We used the IBM SPSS Statistics 23 (https://www.ibm.com/products/spss‐statistics) for statistical analysis. Kolmogorov–Smirnov test was used to assess data distribution. Data without a normal distribution or homogeneity of variance would be compared between groups with nonparametric approach. When comparing qualitative variables, we used Kruskal–Wallis H‐test, ANCOVA, Quade nonparametric ANCOVA and post hoc pairwise comparisons, or Mann–Whitney U test when applicable. Considering the difference in sample size, we additionally reported the effect sizes.^44^ To compare categorical variables between groups, Chi‐square test or Fisher exact test was applied. Age, sex, educational level, and MoCA were controlled as covariates of no interest when comparing neuropsychological scores and graph theoretical metrics.

FDR procedures were applied for the multiple comparisons. A *p* < 0.05 was considered statistically significant.

### 2.8 Sensitivity analysis

#### 2.8.1 Endophenotypes verification

We applied three widely used methods to verify the endophenotypes: receiver operating characteristic (ROC) curve analysis,^12^, ^17^ correlation analysis,^13^ and intraclass correlation coefficient (ICC) analysis.^13^, ^14^

We employed the functional network parameters in the logistic regression and ROC curve analysis to discriminate siblings from healthy controls. High discriminative accuracy indicates that siblings have higher prevalence of functional network abnormalities than the normal population.

We investigated the relation between endophenotype candidates and language test scores. To assess the impact of disease severity and psychiatric comorbidity, we additionally evaluated the correlation of endophenotype candidates with SAS, SDS, age of onset (AOO), disease duration, seizure frequency, and the number of antiseizure medications (ASMs) in patients.

To assess the heritability of potential endophenotypes, we calculated the ICC of graph theory metrics and eFNC between patient‐sibling pairs.^13^, ^14^ One corresponding patient presented bilateral TLE, one failed the fMRI scan due to claustrophobia, and one had poor imaging quality. Hence, there were 10 patient‐sibling pairs in our cohort.

#### 2.8.2 Subgroup analysis

Patients were divided into two groups according to HS and NHS. There were seven unaffected siblings of patients with TLE‐HS (Sib‐HS), and six unaffected siblings of patients with TLE‐NHS (Sib‐NHS). We explored the difference between HC, two patient groups, and two sibling groups.

Besides, differences within LTLE and RTLE subgroups were explored to address the laterality effect.

## 3 RESULTS

### 3.1 Demographic and clinical data

No significant group difference of age, sex, or educational level was noted. But HC performed better in MoCA than patients (*p* = 0.008, FDR‐corrected; *F* = 9.55, *η* ^2^ = 0.089) and siblings (*p* = 0.03, FDR‐corrected; *F* = 14.44, *η* ^2^ = 0.128). Patients had higher anxiety level than HC (*p* = 0.002, FDR‐corrected; *F* = 8.76, *η* ^2^ = 0.085) and siblings (*p* = 0.03, FDR‐corrected; *F* = 7.24, *η* ^2^ = 0.071), and higher depression level than HC (*p* = 0.006, FDR‐corrected; *F* = 11.17, *η* ^2^ = 0.106). The HC group outperformed patients in VFP (*p* = 0.002, FDR‐corrected; *F* = 15.62, *η* ^2^ = 0.142) and BN (*p* = 0.02, FDR‐corrected; *F* = 8.18, *η* ^2^ = 0.080). Details of demographic and clinical data were listed in Table 1.

**TABLE 1:** Demographic and clinical data.

	HC	Sibling	TLE	statistic	*η* ^2^ or Φ	*p* value
*N*	30	13	58	–	–	–
Age, years, median (IQR)	26.0 (18.0)	31.0 (7.0)	29.0 (10.0)	0.87^a^	0.012	0.65
Sex, Male/Female	14/16	3/10	29/29	3.13^b^	0.176	0.21
Education, median (IQR)	12.0 (7.0)	9.0 (5.0)	12.0 (7.0)	1.95^a^	0.001	0.38
MoCA, median (IQR)	29.0 (3.0)	25.0 (5.0)	26.0 (5.0)	11.05^a^	0.092	0.009*
VFC, median (IQR)	24.5 (16.0)	18.0 (12.0)	18.0 (9.0)	3.31^c^	0.066	0.04
VFP, mean (SD)	34.2 (17.6)	23.9 (9.6)	24.4 (13.1)	7.82^d^	0.143	0.004*
BN, median (IQR)	27.0 (5.0)	24.0 (7.0)	25.0 (5.0)	4.09^c^	0.080	0.04*
SAS, median (IQR)	38.0 (8.0)	40.0 (10.0)	45.5 (14.0)	8.82^c^	0.158	0.003*
SDS, mean (SD)	39.4 (10.3)	42.3 (10.5)	48.4 (9.8)	6.04^d^	0.114	0.009*
duration, years, median (IQR)			10.0 (16.0)			
AOO, years, median (IQR)			17.0 (10.0)			
Laterality, Left/Right			25/33			
Febrile convulsion history			3 (5.2%)			
SGS history			46 (79.3%)			
Number of ASMs						
1			36			
2			21			
3			1			
Seizure frequency						
Every year			15			
Every month			21			
Every week			12			
Every day			10			

Abbreviations: AOO, age of onset; ASMs, antiseizure medications; BN, Boston Naming Test; HC, healthy controls; IQR, interquartile range; MoCA, Montreal Cognitive Assessment; SAS, Self‐Rating Anxiety Scale; SD, standard deviation; SDS, Self‐Rating Depression Scale; SGS, secondary generalized seizures; TLE, patients with temporal lobe epilepsy; VFC, verbal fluency character test; VFP, verbal fluency Pinyin test.

^a^ H value of Kruskal–Wallis H‐test.

^b^ χ^2^ value of Chi‐squared test.

^c^ *F* value of Quade nonparametric analysis of covariates.

^d^ *F* value of analysis of covariates.

* *p* values are FDR corrected across nine comparisons. The uncorrected *p* = 0.004, <0.001, 0.02, <0.001, and 0.004, in order.

### 3.2 Task residual‐functional connectivity topology

**FIGURE 1:**
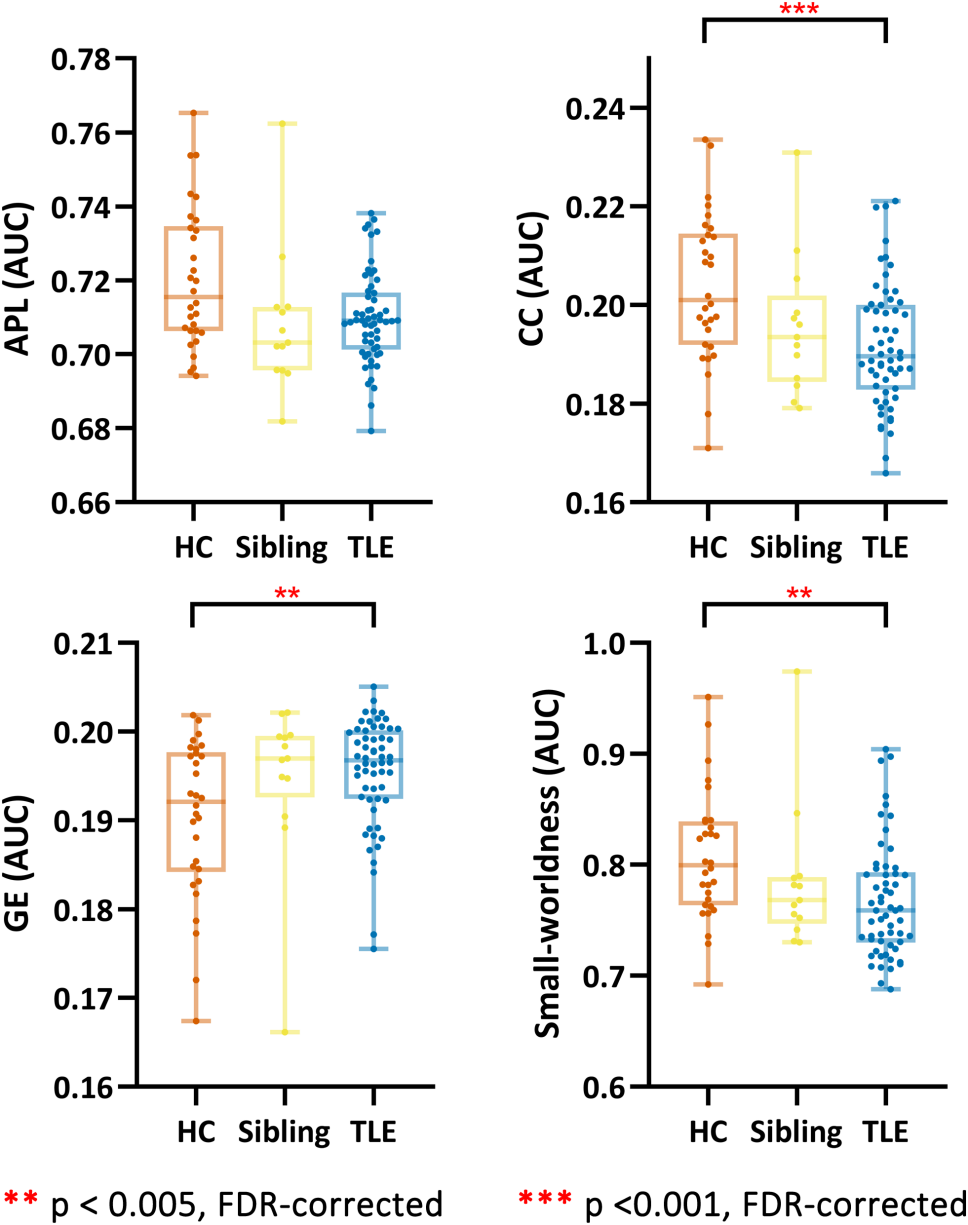
Topological parameters during the rest. The AUC comparison of three groups for APL, CC, GE, and small‐worldness throughout all densities. APL, average shortest path length; AUC, area under curve; CC, clustering coefficiency; GE, global efficiency; HC, healthy controls; TLE, temporal lobe epilepsy.

On the overall scale (Figure 1), patients presented significantly higher GE (*p* = 0.01, FDR‐corrected; *F* = 9.26; *η* ^2^ = 0.090), lower CC (*p* < 0.001, FDR‐corrected; *F* = 15.09; *η* ^2^ = 0.138), and lower σ (*p* = 0.006, FDR‐corrected; *F* = 10.26; *η* ^2^ = 0.098). Though the difference was not statistically significant, siblings showed the same but milder abnormality as patients.

The group difference of functional connectivity topology under specific densities was presented in Figure S1.

### 3.3 Task‐based functional connectivity topology

**FIGURE 2:**
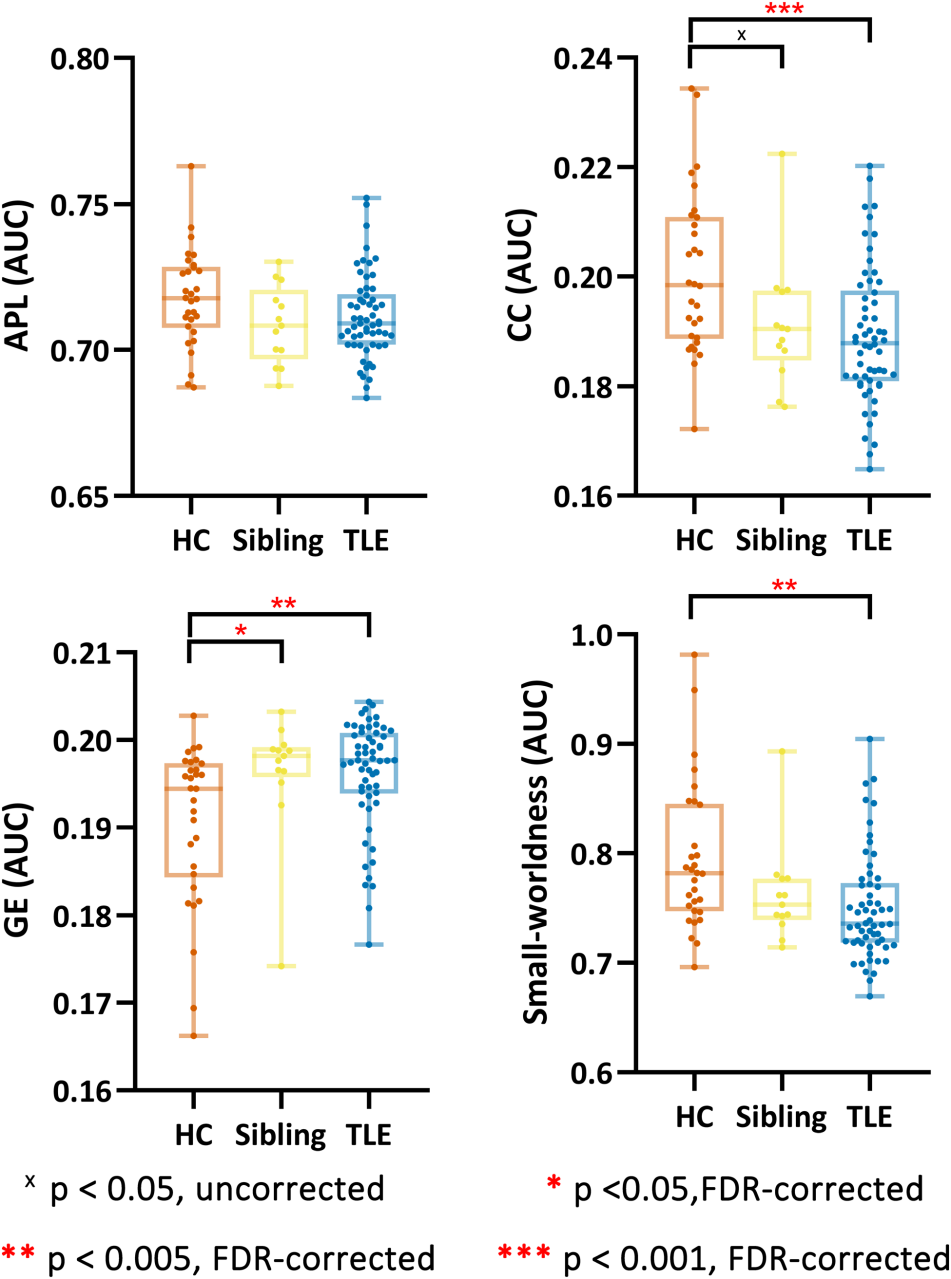
Topological parameters during the task. The AUC comparison of three groups for APL, CC, GE, and small‐worldness throughout all densities. APL, average shortest path length; AUC, area under curve; CC, clustering coefficiency; GE, global efficiency; HC, healthy controls; TLE, temporal lobe epilepsy.

On the overall scale (Figure 2), patients had higher GE (*p* = 0.005, FDR‐corrected; *F* = 10.85; *η* ^2^ = 0.103), lower CC (*p* < 0.001, FDR‐corrected; *F* = 14.30; *η* ^2^ = 0.132), and lower σ (*p* = 0.002, FDR‐corrected; *F* = 12.64; *η* ^2^ = 0.119) in comparison with HC. Compared to HC, siblings also had higher GE (*p* = 0.04, FDR‐corrected; *F* = 5.23; *η* ^2^ = 0.053) and lower CC (*p* = 0.05, uncorrected; *F* = 4.33; *η* ^2^ = 0.044).

The group difference of functional connectivity topology under specific densities was presented in Figure S2.

### 3.4 Group‐ICA and post hoc eFNC

The group‐ICA successfully separated the DMN, limbic system, ventral attention network (VAN), and other task‐related networks. The detailed labeling and presenting of all 20 ICs are listed in Figure S3.

**FIGURE 3:**
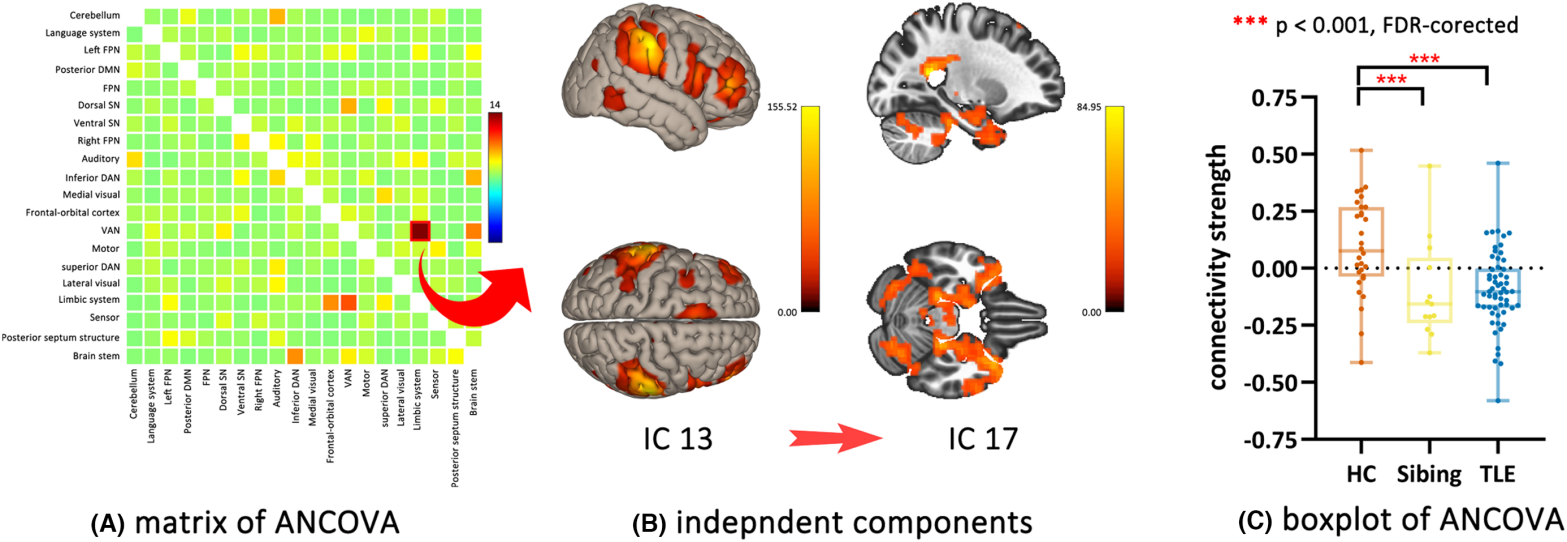
The results of eFNC analysis. (A) ANCOVA matrix of eFNC analysis. the eFNC from the IC 13 to the IC 17 (red squared) was different across three groups (*p* < 0.05, FDR‐corrected); (B) brain regions of IC 13 and IC 17; (C) group comparison of the eFNC from the IC 13 to the IC 17. ANCOVA, analysis of covariates; eFNC, effective functional network connectivity; HC, healthy controls; IC, independent component; TLE, temporal lobe epilepsy.

The effective connectivity from the IC 13 (VAN) to the IC 17 (limbic system) was significantly different across the three groups (*p* < 0.05, FDR‐corrected; *F* = 14.01; *η* ^2^ = 0.230; Figure 3A,B). Post hoc pairwise comparison revealed reduced eFNC in the siblings (*p* < 0.001, FDR‐corrected; *F* = 16.62; *η* ^2^ = 0.150) and patients (*p* < 0.001, FDR‐corrected; *F* = 23.31; *η* ^2^ = 0.199) compared to HC (Figure 3C).

### 3.5 Sensitivity analysis

#### 3.5.1 Endophenotypes verification

**FIGURE 4:**
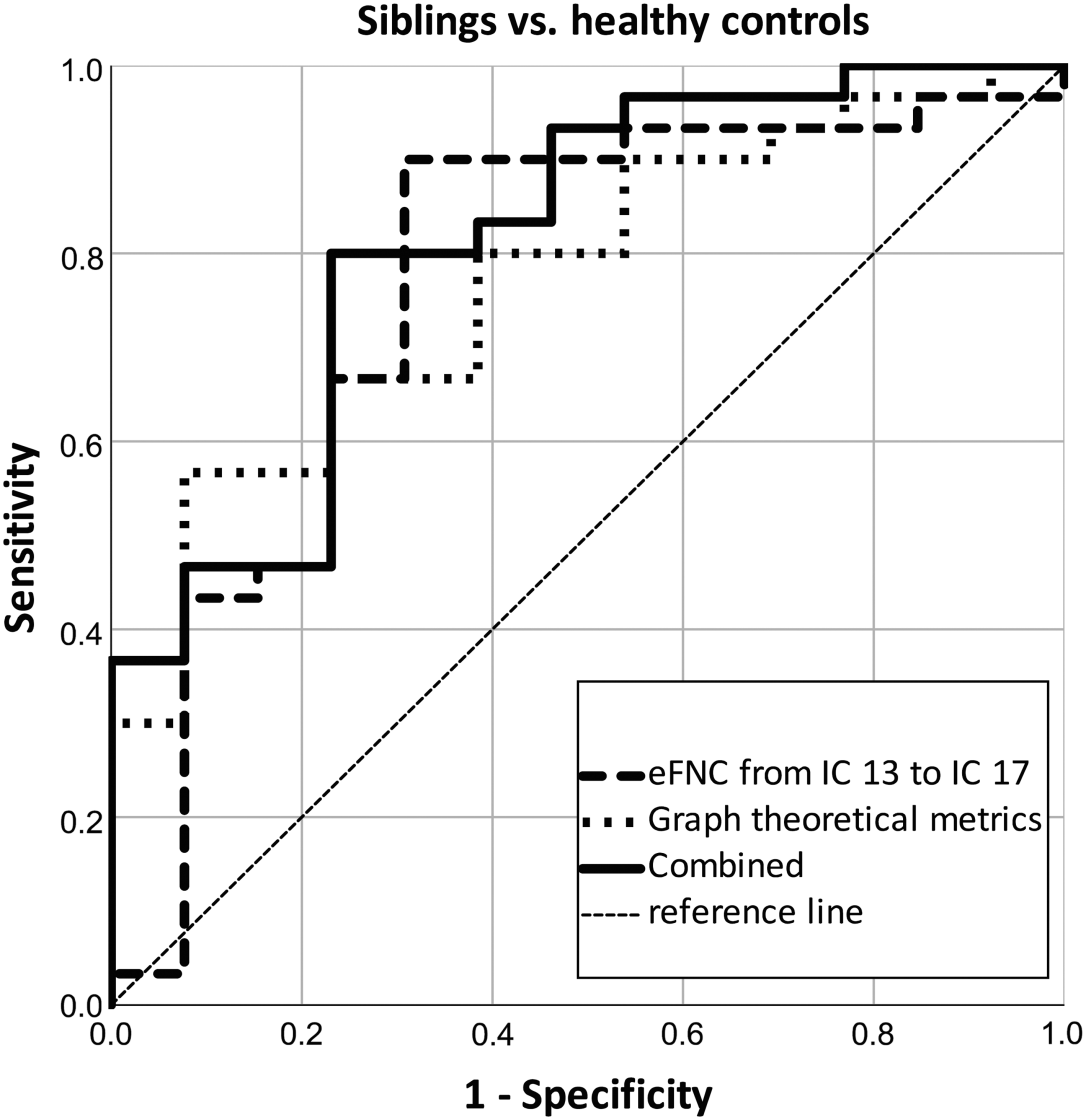
Siblings discriminating using functional abnormalities. eFNC, effective functional network connectivity; IC, independent component.

Both graph theoretical metrics (AUC, 0.767; 95% CI, 0.595–0.938) and the eFNC from the VAN to the limbic system (AUC, 0.772; 95% CI, 0.627–0.917) reached high accuracy when discriminating siblings from HC. The combination of two methods had an AUC = 0.813, 95% CI = 0.672–0.954 (Figure 4).

Graph theory metrics or eFNC were not correlated with disease burden (AOO, disease duration, and seizure frequency), or number of ASMs (*p* > 0.05, uncorrected). Stronger connectivity between the VAN and the limbic system was correlated with higher VFP scores (*p* = 0.02, uncorrected; *r* = 0.32).

GE and CC in tr‐FC and tb‐FC, and σ in tb‐FC were highly correlated between patients with TLE and their unaffected siblings (Table 2).

**TABLE 2:** Intraclass correlation coefficients.

Parameter	ICC	95% CI Lower	95% CI Upper	*p* value
APL_tr‐FC	0.45	−0.21	0.83	0.09
CC_tr‐FC	0.77	0.32	0.94	0.009*
GE_tr‐FC	0.66	0.09	0.90	0.03*
σ_tr‐FC	0.13	−0.51	0.68	0.36
APL_tb‐FC	−0.56	−0.87	0.06	0.96
CC_tb‐FC	0.59	−0.02	0.88	0.03
GE_tb‐FC	0.77	0.31	0.94	0.009*
σ_tb‐FC	0.87	0.57	0.97	0.003*
eFNC	0.26	−0.40	0.75	0.22

Abbreviations: APL, average shortest path length; CC, clustering coefficiency; CI, confident interval; eFNC, effective functional network connectivity; GE, global efficiency; ICC, intraclass correlation coefficient; tb‐FC, task‐based functional connectivity; tr‐FC, task residual‐functional connectivity; σ, small‐worldness.

* *p* values were FDR‐corrected across all parameters. The uncorrected *p* = 0.003, 0.02, 0.003, <0.001, for CC_tr‐FC, GE_tr‐FC, GE_tb‐FC, and σ_tb‐FC separately.

#### 3.5.2 HS and NHS subgroups

Patients with HS and their siblings had relatively worse language performance and more severe topological disturbance than patients without HS and their siblings. The eFNC from the VAN to the limbic system was disrupted at a similar level in patients with and without HS (see details in Material S1, Table S1, Figure S4–S6).

#### 3.5.3 LTLE and RTLE subgroups

The results roughly replicated that in the whole group analyses. Meanwhile, LTLE‐NHS also presented milder but impaired tr‐FC topological features compared to HC (see details in Material S1, Figure S7–S9).

#### 3.5.4 SAS and SDS as covariates of no interest

Since the limbic system is related to psychiatric comorbidity, we examined the group difference of eFNC after controlling for SAS and SDS scores. HC presented stronger functional connectivity compared to patients (*p* < 0.001, FDR‐corrected; *F* = 14.63; *η* ^2^ = 0.186) and siblings (*p* < 0.001, FDR‐corrected; *F* = 16.15; *η* ^2^ = 0.201). In addition, the eFNC strength was not correlated with SAS or SDS in the patients (*p* > 0.05, uncorrected).

#### 3.5.5 Effect of intellectual disability

Since patients and siblings presented lower MoCA scores compared to HC, we calculated the partial correlation between MoCA scores and functional statistics. The MoCA score was not correlated with functional network connectivity and graph statistics (*p* > 0.8).

## 4 DISCUSSION

With a Chinese character version of verbal fluency task, we observed shared functional network abnormalities in patients with TLE and their unaffected siblings. The topological disturbance and network‐level impairment, especially the eFNC from the VAN to the limbic system, could be considered functional endophenotypes that might contribute to the susceptibility of seizures and cognitive decline in TLE.

Verbal fluency decline is a common deficit in TLE. A Chinese character verbal fluency task mobilizes orthographic, tonal, phonological, and semantic processing procedures.^32^ The Chinese character verbal fluency task triggered widespread activation and deactivation effects, including the deactivation of the DMN and the activation of task‐related networks, ensuring an ideal tool for evaluating language decline and network disruption.^31^

By exploring the graph theory metrics, we observed a less small‐world and more random topology in patients with TLE and their unaffected siblings. A small‐world network balances information local processing and global transferring, with economical energy cost.^45^ This property was impaired, indicating reduced brain efficiency in patients and their unaffected siblings.^23^, ^24^, ^25^, ^26^ We also found shared lower CC in patients and their unaffected siblings, representing a reduced local information processing ability.^20^, ^23^, ^26^ In TLE, disconnection within local networks such as the left temporal lobe language network^46^ and DMN^47^ impeded local information processing and resulted in language decline. While the ictal network bears a more regular topology,^48^, ^49^ the interictal functional network would be a more random network.^20^ Our study supported this observation as patients and their unaffected siblings showed lower CC and higher GE. Neuronal synchronization is an important mechanism underlies seizure generation and propagation.^50^, ^51^, ^52^ A random functional network has lower threshold for synchronization.^49^, ^53^ In the mathematic hippocampal model established by Netoff et al., with the increasing of random connections, the network had lower threshold for synchronization, and the neural activity shifted to seizure‐like activities.^54^ These indicated a predisposed susceptibility of seizures in patients with TLE and their unaffected siblings.

In addition, we explored the eFNC between networks identified by group‐ICA. In patients and their unaffected siblings, we observed reduced connectivity from the VAN to limbic system. The VAN served to regulate one's attention when unexpected stimuli appeared during a task.^55^ Along with hippocampi, the limbic system was at the center of seizure generation. Previous studies had observed macrostructural^56^, ^57^ and microstructural^58^ damage of the limbic system in TLE. Girardi‐Schappo et. la. observed slower information flow in and out the limbic system in TLE, which was partially controlled by hippocampal volume.^10^ Another independent study also reported disrupted hierarchical organization originated from the limbic system. The severity of organization alteration was correlated with cognitive performance.^29^ We observed a positive correlation between the language test performance and the connectivity strength. Our findings suggested that the disconnection between the VAN and the limbic system during tasks could had contributed to the language impairment. This indicated that, at least in some cases, the cognitive decline in patients with TLE and their unaffected siblings^59^ was genetically predisposed.

The genetic background of seizure and cognitive decline in TLE is a topic of ongoing debate. While structural abnormalities connected directly to the genotype that influence cortex development, functional disturbances lie closer to seizure generation and spreading, representing an essential step from genotype to phenotype.^12^, ^17^ A recent resting‐state electroencephalography‐fMRI study noted impaired ability to suppress sensorimotor activities in patients with TLE and their unaffected relatives.^18^ We reported two novel functional features shared by patients with TLE and their unaffected siblings. These features may not be the consequence of seizures but were likely to be predisposed traits determined by shared genetic and (or) environmental factors. To explore their potential as functional endophenotypes, we conducted three analyses. The ROC curve analyses demonstrated a higher prevalence of functional network impairment in unaffected siblings of patients than in the normal population. ICC analyses showed high consistency between patients and their siblings, further suggesting the genetic background of functional network abnormalities. Also, topological parameters and eFNC were not related to disease burden. These results suggested that the graph theoretical metrics and eFNC conformed to the definitions of endophenotypes,^60^ and were connected to TLE at the population level. Our findings indicated that the disruption of functional connectivity in TLE were prior to and might promote the onset of seizures and cognitive impairment under certain environmental factors. Unaffected siblings did not develop epilepsy despite bearing a genetic susceptibility, potentially indicating that additional environmental factors are needed to cause seizures. Alternatively, milder topological abnormalities in siblings may not be sufficient to induce epilepsy. We also provided candidates for functional endophenotypes, which can be used as quantitative traits in future genetic searching, improving the success and power of such studies.

We conducted sensitivity analyses in subgroups to explore the effect of HS and lesion laterality. Patients with HS and their siblings presented more severe topological disturbance compared to patients without HS and their siblings. This observation not only further supported the heritability of network topology but also suggested that HS was related to whole‐brain network topology. A possible explanation is that HS is a protruding phenotype of genetic factors that affect whole‐brain development.^14^ Alternatively, genetic factors may only lead to the maldevelopment of the hippocampus,^5^ and the whole‐brain functional alteration is a downstream event of HS. Did not differ between patients with and without HS, the eFNC from the VAN to the limbic system may be a universal feature independent to hippocampal pathologies in TLE. Meanwhile, patients with LTLE and patients with RTLE had similar functional network patterns, indicating that lesion laterality was not a confounder. Also, patients had relatively lower MoCA score compared to HC. Hence, we defined MoCA scores as a covariate of no interest in group comparison and correlation analysis. Also, MoCA scores was not correlated with function statistics.

There are limitations. First, our cohort of siblings was relatively small. Hence, we collected clinical details and applied well‐established and robust methods. Even though the power of analyses for unaffected siblings was lowered by the small sample size, we still observed significant differences between siblings and healthy controls, suggesting our results are reliable. Further sensitivity analyses also verified that the functional network abnormalities are promising endophenotypes. Still, the results may not be representative, and a larger cohort of unaffected siblings would benefit future studies. Second, some patients had HS despite normal hippocampal volume and MRI signal.^61^ Though we designed a thorough procedure to distinguish HS, missed diagnosis in MRI‐negative patients shall not be ignored. However, our cohort would represent patients with apparent HS, who might have more profound genetic backgrounds for HS and TLE. Third, potential admission rate bias should be admitted since our study was based on subjects from Xiangya Hospital. Before generalizing our results, they should be tested in external data.

In conclusion, we observed shared whole‐brain functional network topological disruption and impaired eFNC from the VAN to the limbic system in patients with TLE and their unaffected siblings. The functional disturbance could be considered endophenotypes that precede and even promote seizure susceptibility and cognitive decline in TLE. These imaging traits could be applied as predictors for seizure onset and language decline in future prospective studies. Our findings would also benefit future genetic searching aimed for common variants in TLE.

## AUTHOR CONTRIBUTIONS

Kangrun Wang designed and conceptualized study, played a role in the acquisition of data, analyzed the data and functional connectivity, and drafted the manuscript. Fangfang Xie designed and conceptualized study, analyzed the data, major role in the acquisition of data, and drafted the manuscript. Charong Liu, Ge Wang, and Min Zhang analyzed the data and played a role in the acquisition of data. Jialinzi He and Langzi Tan set the parameters of the task and played a role in the acquisition of data. Haiyun Tang played a role in the acquisition of data. Fenghua Chen and Bo Xiao designed and conceptualized study. Yanmin Song supervised the study, played a role in the acquisition of data, and revised the final manuscript. Lili Long designed and conceptualized the study, supervised the study, and revised the final manuscript.

## CONFLICT OF INTEREST

The authors declare that they have no conflict of interest.

## INFORMED CONSENT

Written informed consent was obtained from all participants.

## Supporting information


Appendix S1

## Data Availability

The data that support the findings of this study are available from the corresponding author upon reasonable request.
