# Supplementary material for: Shared functional network abnormality in patients with temporal lobe epilepsy and their siblings
Source: CNS Neurosci Ther. 2023 Jan 17;29(4):1109–19. doi: 10.1111/cns.14087 (PMC10018100; doi:10.1111/cns.14087)
Supplement: Supplementary file 1 — Appendix S1 [file CNS-29-1109-s001.docx]

**Supplementary Material**

**for**

**Shared functional network abnormality in patients with TLE and their unaffected siblings**

**1. HS and NHS subgroups**

**1.1. Demographic and clinical data**

No group difference of age, sex, or educational level was noted. HC significantly outperformed both patient groups in the SAS (p = 0.009, FDR-corrected; F = 10.40; η^2^ = 0.102 for HC vs. TLE-HS and p = 0.009, FDR-corrected; F = 11.20; η^2^ = 0.109 for HC vs. TLE-NHS) and MoCA (p = 0.05, FDR-corrected; F =7.34; η^2^ = 0.074 for HC vs. TLE-HS and p = 0.05, FDR-corrected; F = 6.89; η^2^ = 0.070 for HC vs. TLE-NHS). HC also had statistically better performance in SDS (p = 0.02, FDR-corrected; F = 10.97; η^2^ = 0.107) and VFP (p = 0.002, FDR-corrected; F = 16.18; η^2^ = 0.150) than TLE-HS. Except for lower MoCA in Sib-HS compared to HC (*p* = 0.05, FDR-corrected; F = 6.03; η^2^ = 0.061), siblings did not statistically differ from HC in neuropsychological tests. Patients and siblings presented poorer performance than HC in BN and VFC, though the difference was not statistically significant (Table S1).

In terms of clinical data, the two patient groups did not differ in the AOO, disease duration, seizure frequency, lesion laterality, history of febrile convulsion, number of ASMs, and seizure types.

**1.2. Task-residual functional connectivity topology**

Significant differences have been detected between TLE-HS and HC groups in GE (*p* = 0.02, FDR-corrected; F = 10.75; η^2^ = 0.101), CC (*p* < 0.001, FDR-corrected; F = 20.67; η^2^ = 0.177), and σ (*p* < 0.001, FDR-corrected; F = 17.86; η^2^ = 0157). TLE-NHS showed lower CC (*p* = 0.04, FDR-corrected; F = 6.02; η^2^ = 0.059) and higher σ (p = 0.04, uncorrected; F = 4.70; η^2^ = 0.047) compared to HC. TLE-HS also had lower CC (*p* = 0.05, uncorrected; F = 4.05; η^2^ = 0.041) and higher σ (p = 0.05, uncorrected; F = 3.94; η^2^ = 0.039) compared to TLE-NHS (Fig. S4).

Sib-HS showed a pattern similar to TLE-HS as they presented higher GE (*p* = 0.03, FDR-corrected; F = 8.16; η^2^ = 0.078), lower CC (*p* = 0.03, FDR-corrected; F = 7.91; η^2^ = 0.076), and higher σ (*p* = 0.04, uncorrected; F = 4.77; η^2^ = 0.047) compared to HC. Compared to Sib-NHS, Sib-HS exhibited a trend for lower CC (*p* = 0.05, uncorrected; F = 3.94; η^2^ = 0.039). There was no significant difference between HC and Sib-NHS (Fig. S4).

**1.3. Task-based functional connectivity topology**

Compared to other two groups, TLE-HS patients showed significantly lower CC (*p* < 0.001, FDR-corrected; F = 19.96; η^2^ = 0.172 for HC vs. TLE-HS and *p* = 0.05, FDR-corrected; F = 5.21; η^2^ = 0.051 for TLE-NHS vs. TLE-HS) and lower σ (*p* < 0.001, FDR-corrected; F =22.17; η^2^ = 0.188 for HC vs. TLE-HS and *p* = 0.05, FDR-corrected; F = 5.95; η^2^ = 0.058 for TLE-NHS vs. TLE-HS). TLE-HS also had significantly higher GE than HC (*p* = 0.003, FDR-corrected; F = 14.68; η^2^ = 0.133). Meanwhile, TLE-NHS patients exhibited higher GE (*p* = 0.03, uncorrected; F = 4.97; η^2^ = 0.049), lower CC (*p* = 0.04, uncorrected; F = 4.44; η^2^ = 0.044), and lower σ (*p* = 0.04, uncorrected; F = 4.78; η^2^ = 0.047) comparing with HC (Fig. S5).

Same as TLE-HS, Sib-HS presented lower CC (*p* = 0.03, FDR-corrected; F = 8.66; η^2^ = 0.083 for HC vs. Sib-HS and *p* = 0.05, FDR-corrected; F = 5.40; η^2^ = 0.053 for Sib-NHS vs. Sib-HS) and σ (p = 0.03, FDR-corrected; F = 7.45; η^2^ = 0.072 for HC vs. Sib-HS and p = 0.05, FDR-corrected; F = 5.42; η^2^ = 0.053 for Sib-NHS vs. Sib-HS) than other two groups. Sib-HS also showed higher GE (p = 0.04, FDR-corrected; F = 7.59; η^2^ = 0.073) compared to HC. There was no significant difference between HC and Sib-NHS (Fig. S5).

**1.4. Group-ICA and post hoc FNC**

The effective connectivity from the VAN to the limbic system was significantly different across five groups (*p* < 0.05, FDR-corrected). Post hoc pairwise comparison revealed stronger FNC in the HC compared to other four groups (*p* = 0.03, 0.004, 0.002, 0.008, FDR-corrected; F = 6.96, 12.57, 15.98, 9.94; η^2^ = 0.070, 0.120, 0.148, 0.097 for HC vs. Sib-HS, Sib-NHS, TLE-HS, and TLE-NHS, separately; Fig. S6).

**2. LTLE and RTLE subgroups**

**2.1. Task-residual functional connectivity topology**

Compared to HC, LTLE-HS and LTLE-NSH both showed higher GE (*p* = 0.05, FDR-corrected; F = 5.19; η^2^ = 0.098 for HC vs. LTLE-HS and *p* = 0.05, FDR-corrected; F = 5.52; η^2^ = 0.103 for HC vs. LTLE-NHS), lower CC (*p* = 0.02, FDR-corrected; F = 9.146; η^2^ = 0.160 for HC vs. LTLE-HS and *p* = 0.04, FDR-corrected; F = 5.69; η^2^ = 0.106 for HC vs. LTLE-NHS) and lower σ (*p* = 0.05, FDR-corrected; F = 6.18; η^2^ = 0.114 for HC vs. LTLE-HS and *p* = 0.05, uncorrected; F = 4.14; η^2^ = 0.079 for HC vs. LTLE-NHS) comparing with HC (Fig. S7A). Also, LTLE-HS presented lower APL compared to HC (*p* = 0.05, FDR-corrected; F = 6.40; η^2^ = 0.118).

Compared to HC (Fig. S8A), RTLE-HS presented significantly higher GE (*p* = 0.03, FDR-corrected; F = 7.72; η^2^ = 0.114), lower CC (*p* = 0.004, FDR-corrected; F = 11.56; η^2^ = 0171) and lower σ (*p* = 0.008, FDR-corrected; F = 10.17; η^2^ = 0.154).

**2.2. Task-based functional connectivity topology**

LTLE-HS exhibit higher GE (*p* = 0.05, FDR-corrected; F = 6.59; η^2^ = 0.121), lower CC (*p* = 0.009, FDR-corrected; F = 9,92; η^2^ = 0.171), and lower σ (*p* = 0.03, FDR-corrected; F = 7.34; η^2^ = 0.133) comparing with HC in tb-FC (Fig. S7B).

Meanwhile, we observed significant differences between RTLE-HS and HC groups in GE (*p* = 0.007, FDR-corrected; F = 10.18; η^2^ = 0.145), CC (*p* = 0.004, FDR-corrected; F = 6.80; η^2^ = 0.108), and σ (*p* = 0.005, FDR-corrected; F = 11.65; η^2^ = 0.172; Fig. S8B).

**2.3. Group-ICA and post hoc FNC**

LTLE-HS (*p* = 0.004, FDR-corrected; F = 12.13; η^2^ = 0.202) and LTLE-NHS (*p* = 0.02, FDR-corrected; F = 7.81; η^2^ = 0.140) presented lower task-modulated effective connectivity from the VAN to the limbic system compared to HC (Fig. S9A).

Compared to HC, the effective connectivity from the VAN to the limbic system was impaired in RTLE-HS (*p* = 0.004, FDR-corrected; F = 11.69; η^2^ = 0.173) and RTLE-NHS (*p* = 0.02, FDR-corrected; F = 7.23; η^2^ = 0.114 ; Fig. S9B).

**3. Generation procedure of figures**

All images in this article were processed with Adobe Photoshop CS5 (<https://www.alivelearn.net/xjview/download/>). Image manipulation only included the arrangement of panels, adding words, and adding symbols.

Brain maps and the comparison matrix are generated by CONN v.20.b (<http://www.nitrc.org/projects/conn>). Figure of ROC (Figure 4) was generated by IBM SPSS Statistics 23 (<https://www.ibm.com/products/spss-statistics>). Other images were generated by GraphPad Prism v8.0.1 (<https://www.graphpad.com/scientific-software/prism/>).


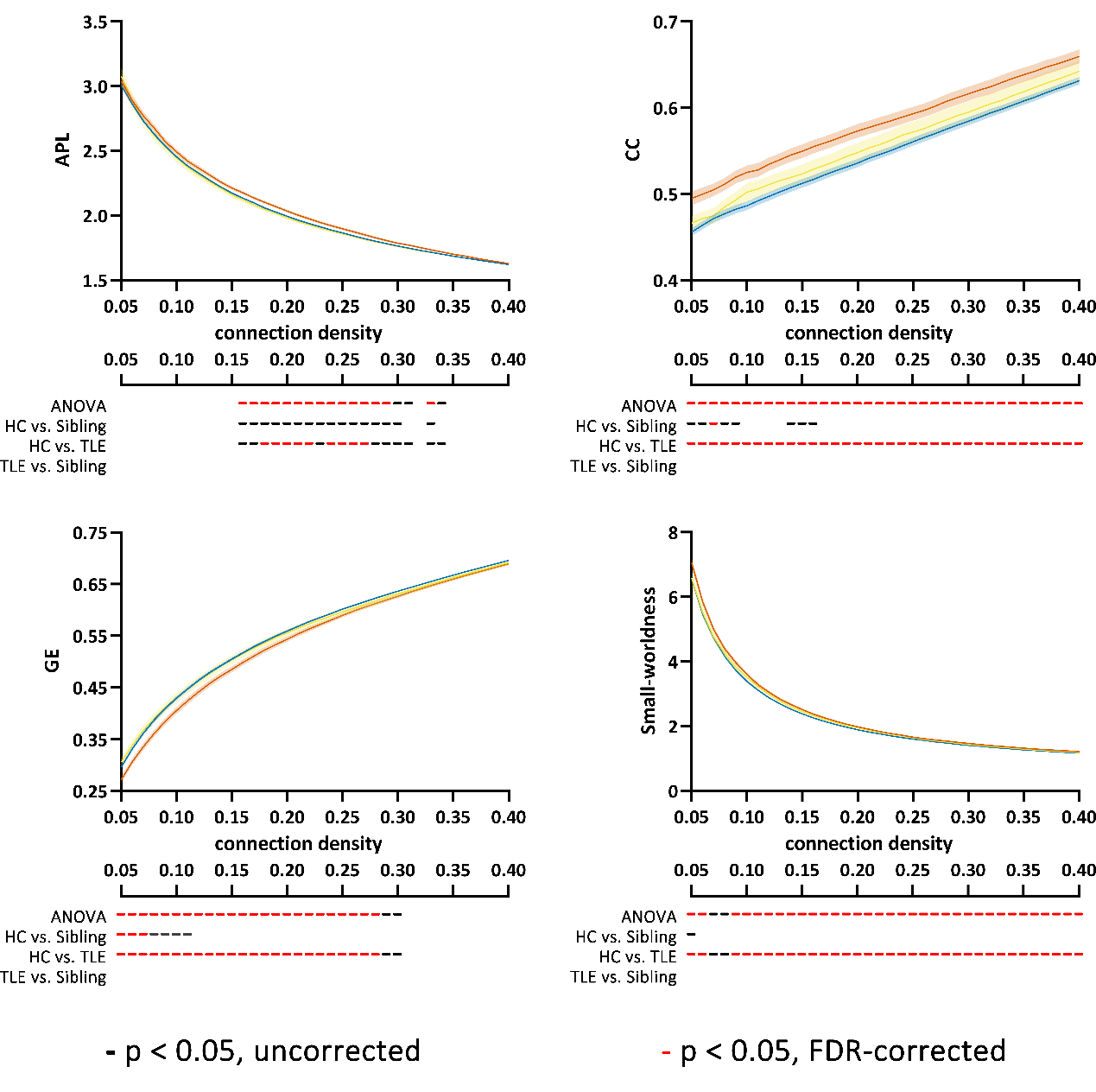


**Fig. S1 Topological parameters during the rest**

Four panels presented the APL, CC, GE, and small-worldness of three groups. For each panel, the line chart on the up shows the parameters across all connectivity densities; and the dotted lines on the low presented the results of group comparison of parameters at certain connectivity density.


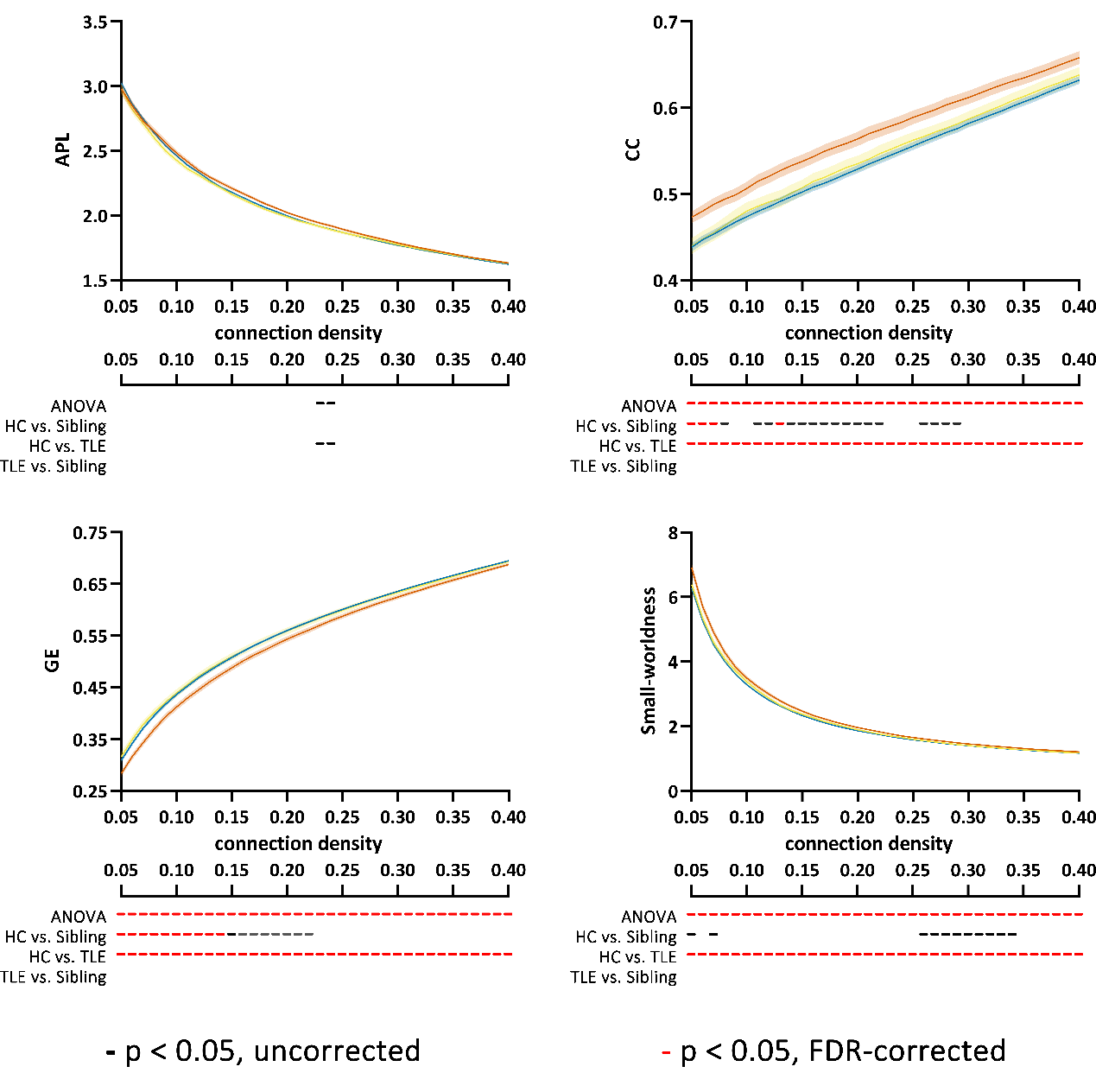


**Fig. S2 Topological parameters during the task**

Four panels presented the APL, CC, GE, and small-worldness of three groups. For each panel, the line chart on the up shows the parameters across all connectivity densities; and the dotted lines on the low presented the results of group comparison of parameters at certain connectivity density.


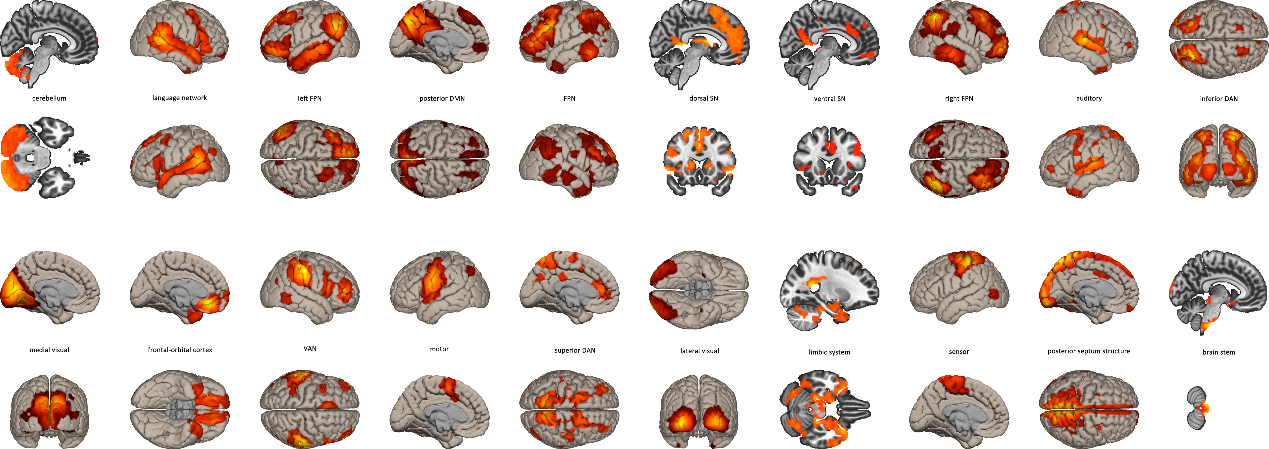


**Fig. S3 Presentation and labelling of 20 independent components**

**
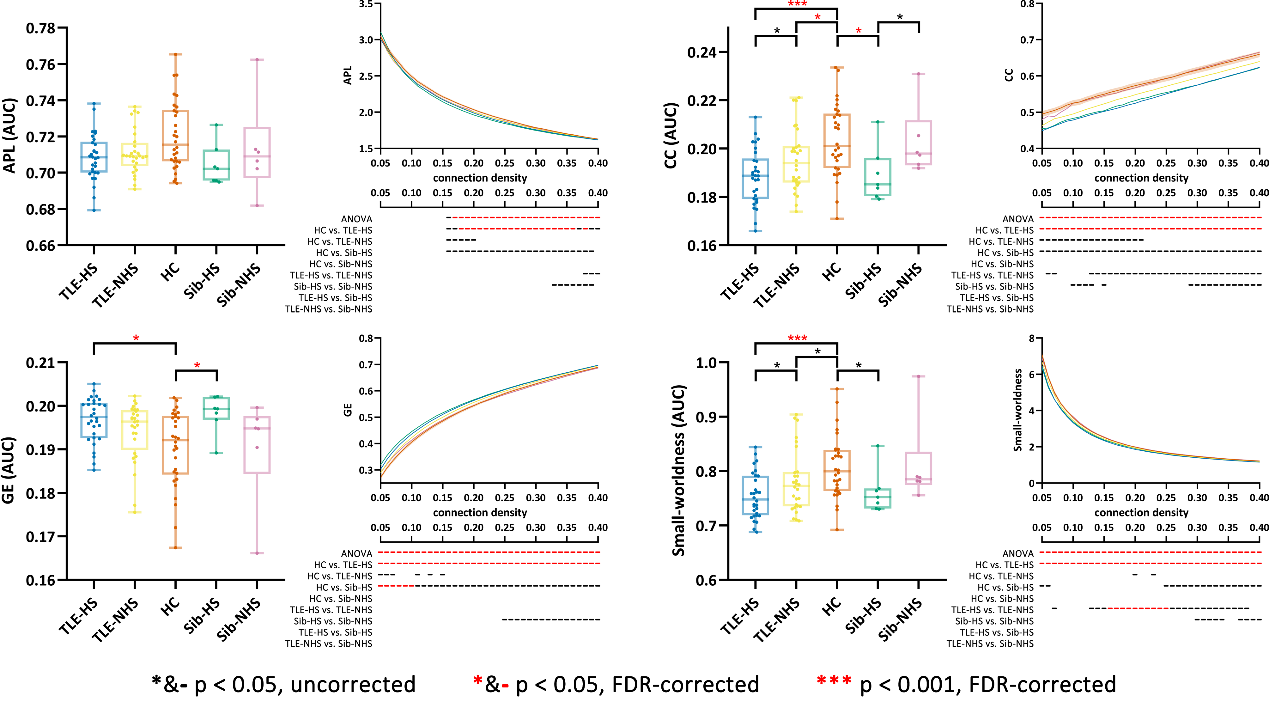
**

**Fig. S4. Topological parameters in hippocampal sclerosis-related sensitivity analysis during the rest**

Four panels presented the APL, CC, GE, and small-worldness of three groups. For each panel, the boxplot chart on the left shows the group comparison of AUC; the line chart on the upper right shows the parameters across all connectivity densities; and the dotted lines on the lower right presented the results of group comparison of parameters at certain connectivity density.

APL, average shortest path length; AUC, area under curve; CC, clustering coefficiency; GE, global efficiency; HC, healthy controls; Sib-HS, unaffected siblings of TLE-HS; Sib-NHS, unaffected siblings of TLE-NHS; TLE-HS, patients with temporal lobe epilepsy and hippocampal sclerosis; TLE-NHS, patients with temporal lobe epilepsy and without hippocampal sclerosis.


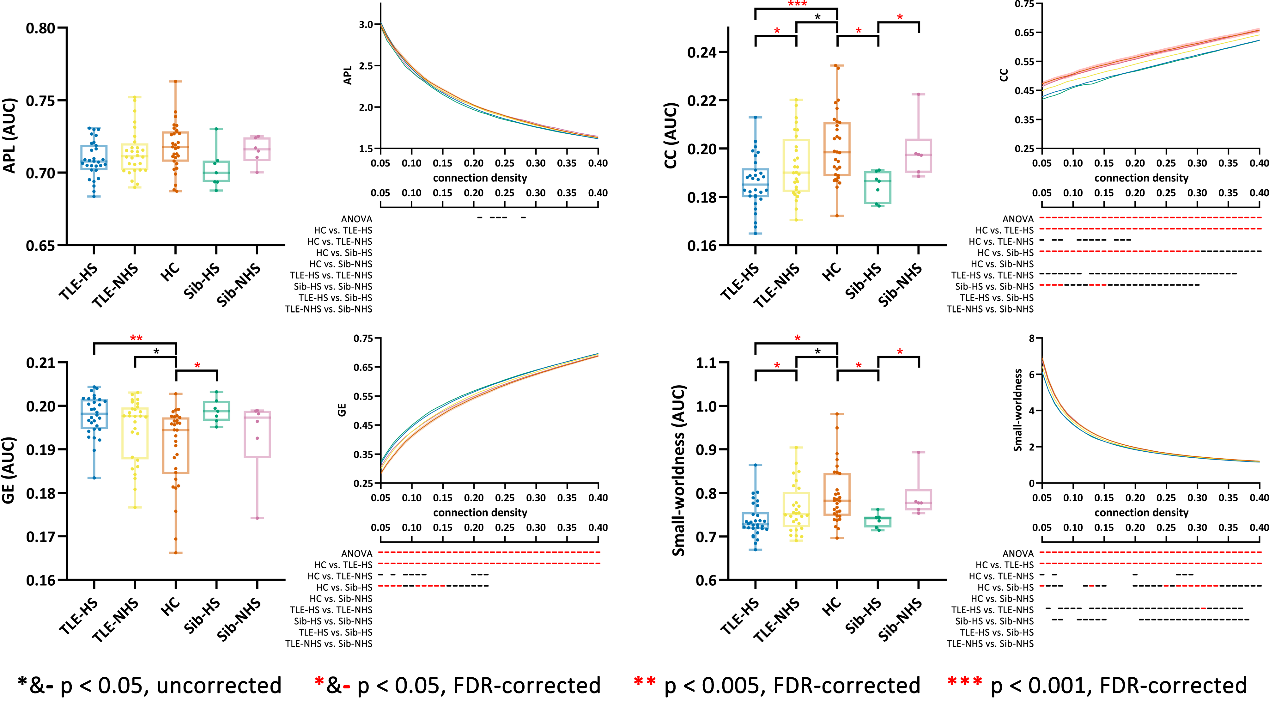


**Fig. S5 Topological parameters in hippocampal sclerosis-related sensitivity analysis during the task**

Four panels presented the APL, CC, GE, and small-worldness of three groups. For each panel, the boxplot chart on the left shows the group comparison of AUC; the line chart on the upper right shows the parameters across all connectivity densities; and the dotted lines on the lower right presented the results of group comparison of parameters at certain connectivity density.

APL, average shortest path length; AUC, area under curve; CC, clustering coefficiency; GE, global efficiency; HC, healthy controls; Sib-HS, unaffected siblings of TLE-HS; Sib-NHS, unaffected siblings of TLE-NHS; TLE-HS, patients with temporal lobe epilepsy and hippocampal sclerosis; TLE-NHS, patients with temporal lobe epilepsy and without hippocampal sclerosis.

**
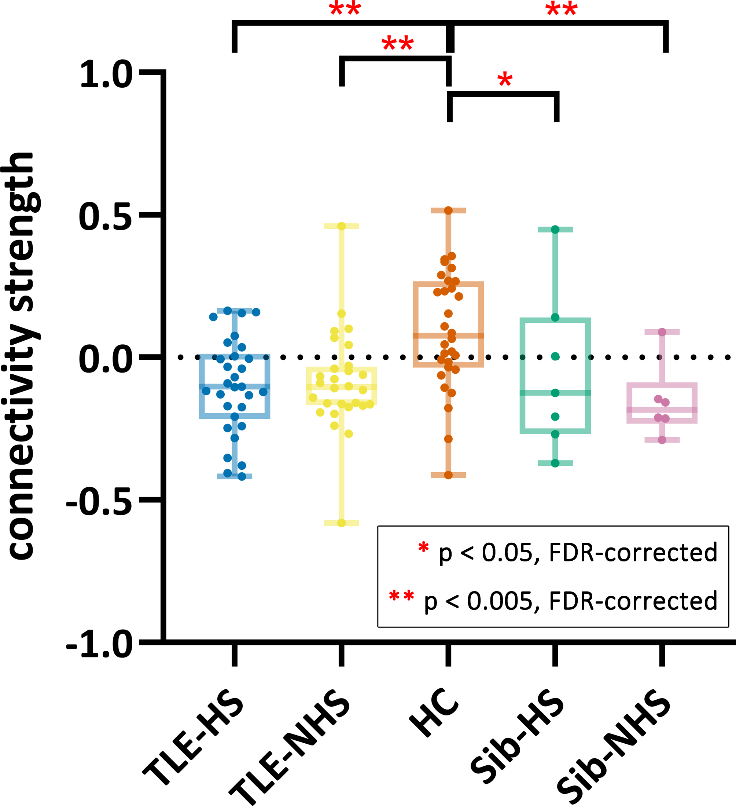
**

**Fig. S6. The results of eFNC analysis in hippocampal sclerosis-related sensitivity analysis**

eFNC, effective functional connectivity; HC, healthy controls; Sib-HS, unaffected siblings of TLE-HS; Sib-NHS, unaffected siblings of TLE-NHS; TLE-HS, patients with temporal lobe epilepsy and hippocampal sclerosis; TLE-NHS, patients with temporal lobe epilepsy and without hippocampal sclerosis.


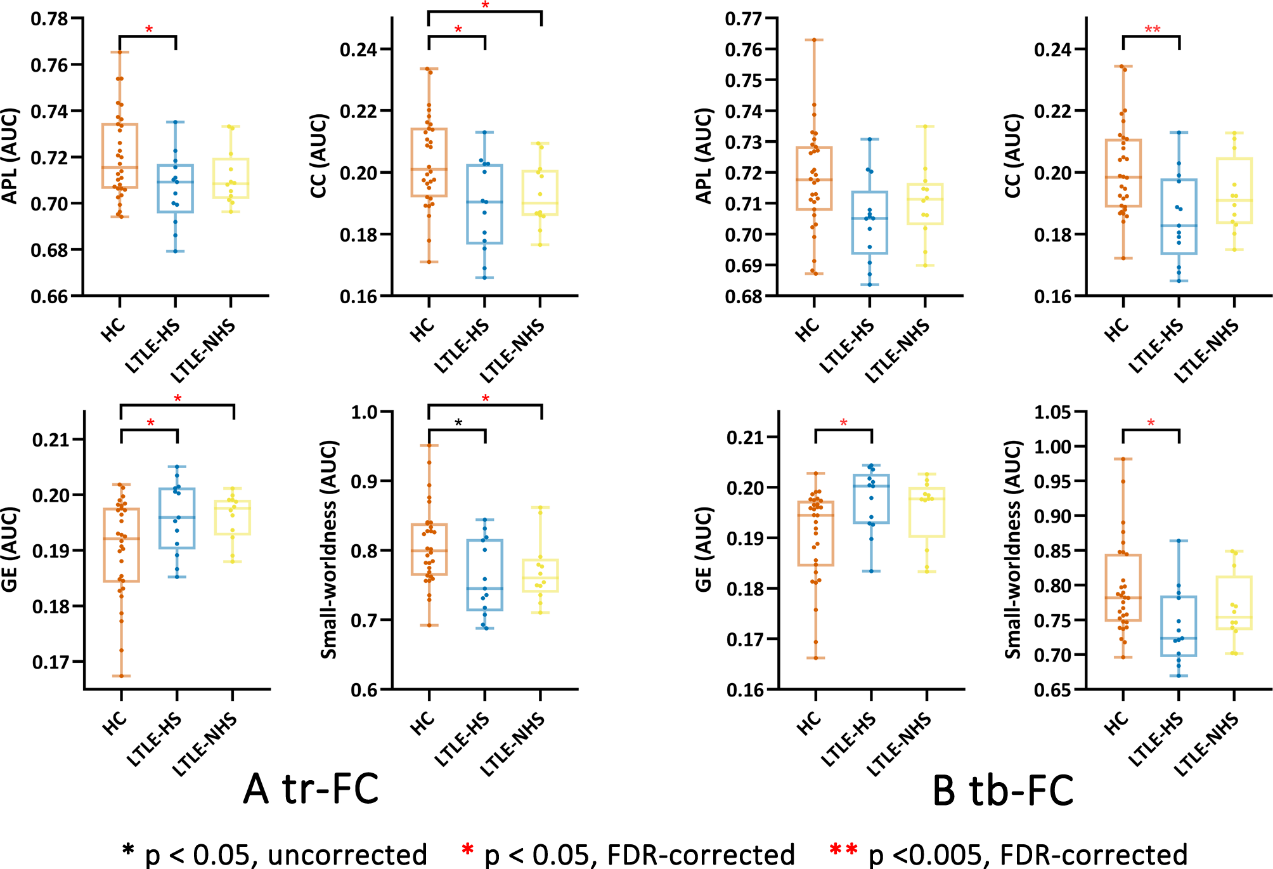


**Fig. S7 Topological parameters in left temporal lobe epilepsy subgroups**

A) during the rest; B) during the task.

APL, average shortest path length; AUC, area under curve; CC, clustering coefficiency; GE, global efficiency; HC, healthy controls; LTLE-HS, patients with left temporal lobe epilepsy and hippocampal sclerosis; LTLE-NHS, patients with left temporal lobe epilepsy and without hippocampal sclerosis.


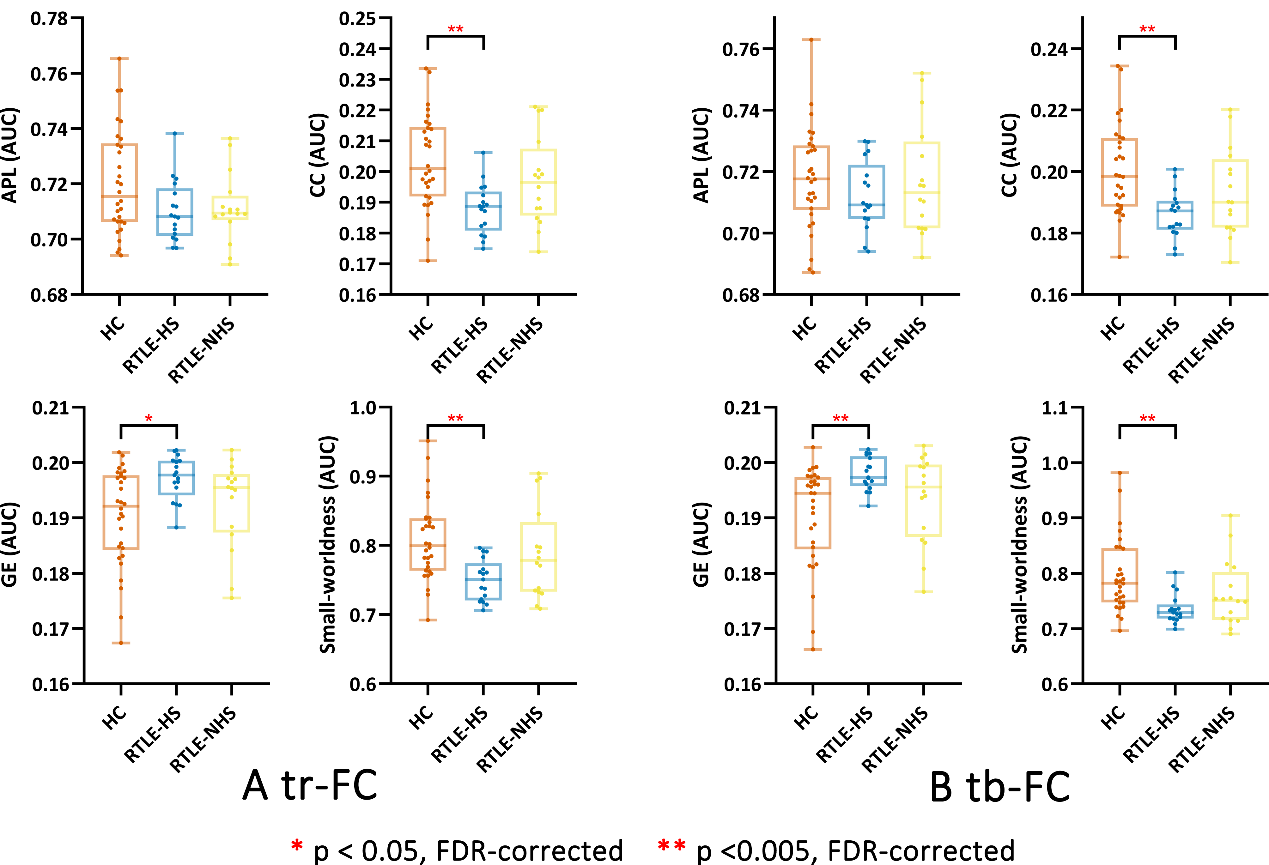


**Fig. S8 Topological parameters in right temporal lobe epilepsy subgroups**

A) during the rest; B) during the task.

APL, average shortest path length; AUC, area under curve; CC, clustering coefficiency; GE, global efficiency; HC, healthy controls; RTLE-HS, patients with right temporal lobe epilepsy and hippocampal sclerosis; RTLE-NHS, patients with right temporal lobe epilepsy and without hippocampal sclerosis.


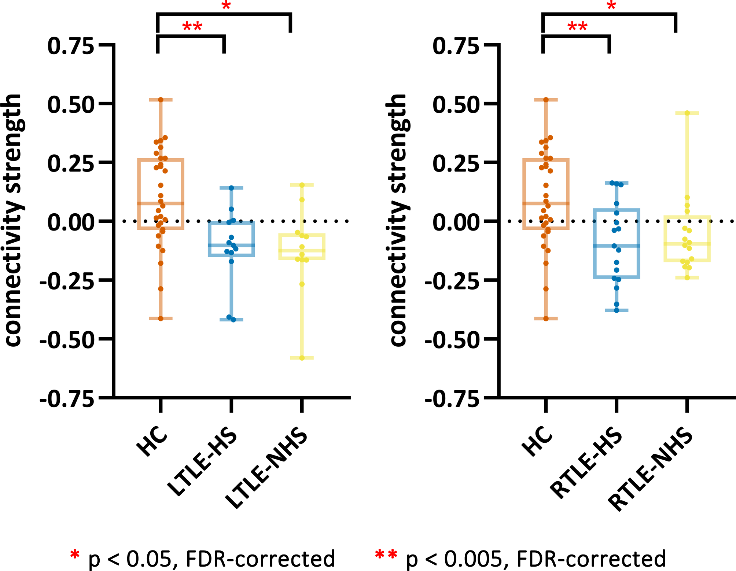


**Fig. S9 The results of eFNC analysis in LTLE and RTLE subgroups.**

eFNC, effective functional connectivity; HC, healthy controls; LTLE, left temporal lobe epilepsy; LTLE-HS, patients with LTLE and hippocampal sclerosis; LTLE-NHS, patients with LTLE and without hippocampal sclerosis; RTLE, right temporal lobe epilepsy; RTLE-HS, patients with RTLE and hippocampal sclerosis; RTLE-NHS, patients with RTLE and without hippocampal sclerosis.

**Table S1 Demographic and clinical data**

|  | HC | Sib-HS | Sib-NHS | TLE-HS | TLE-NHS | Statistic | η^2^ _or_ Φ | P value |
| --- | --- | --- | --- | --- | --- | --- | --- | --- |
| N | 30 | 7 | 6 | 30 | 28 | - | - | - |
| Age, y, median (IQR) | 26.0 (18.0) | 32.0 (6.0) | 30.5 (20.0) | 28.5 (9.0) | 30.0 (12.0) | 2.72^a^ | 0.013 | 0.61 |
| Sex, Male/Female | 14/16 | 1/5 | 2/4 | 14/16 | 15/13 | -^b^ | 0.205 | 0.38 |
| Education, median (IQR) | 12.0 (7.0) | 13.0 (6.0) | 10.5 (7.0) | 10.5 (7.0) | 12.0 (4.0) | 5.83^a^ | 0.019 | 0.22 |
| MoCA , median (IQR) | 29.0 (3.0) | 25.0 (6.0) | 26.5 (5.0) | 26.0 (5.0) | 26.0 (6.0) | 11.71^a^ | 0.08 | 0.03 |
| VFC, median (IQR) | 24.5 (16.0) | 14.0 (6.0) | 22.0 (13.0) | 17.5 (9.0) | 18.5 (9.0) | 2.01^c^ | 0.077 | 0.10 |
| VFP, median (IQR) | 35.5 (28.0) | 22.0 (14.0) | 27.5 (19.0) | 20.0 (17.0) | 27.0 (21.0) | 4.51^c^ | 0.158 | 0.003 |
| BN, median (IQR) | 27.0 (5.0) | 22.0 (5.0) | 24.5 (6.0) | 24.5 (6.0) | 26.0 (5.0) | 2.00^c^ | 0.077 | 0.11 |
| SAS, median (IQR) | 38.0 (8.0) | 43.0 (11.0) | 36.0 (8.0) | 45.5 (13.0) | 46.5 (16.0) | 5.01^c^ | 0.173 | 0.001 |
| SDS, median (IQR) | 39.0 (10.0) | 47.5 (23.0) | 43.5 (21.0) | 50.0 (10.0) | 46.5 (16.0) | 3.24^c^ | 0.119 | 0.02 |
| duration, y, median (IQR) | - | - | - | 9.5 (14.0) | 10.0 (17.0) | -0.413^d^ | 0.003 | 0.68 |
| AOO, y, median (IQR) | - | - | - | 17.0 (10.0) | 17.5 (11.0) | -0.359^d^ | 0.002 | 0.72 |
| Laterality, Left/Right | - | - | - | 13/17 | 12/16 | 0.00^e^ | 0 | 0.98 |
| Febrile convulsion history | - | - | - | 2 (6.7%) | 1 (3.6%) | -^b^ | 0.053 | 1.00 |
| SGS history |  |  |  | 21 (70.0%) | 25 (89.3%) | 3.28^e^ | 0.180 | 0.08 |
| Number of ASMs |  | - | - |  |  | -^b^ | 0.157 | 0.23 |
| 1 | - | - | - | 16 | 20 |  |  |  |
| 2 | - | - | - | 13 | 8 |  |  |  |
| 3 | - | - | - | 1 | 0 |  |  |  |
| Seizure frequency | - | - | - |  |  | -^b^ | 0.128 | 0.69 |
| Every year | - | - | - | 7 | 8 |  |  |  |
| Every month | - | - | - | 13 | 8 |  |  |  |
| Every week | - | - | - | 6 | 6 |  |  |  |
| Every day |  |  |  | 4 | 6 |  |  |  |

a, H value of Kruskal-Wallis H test;

b, no test statistic for Fisher exact test;

c, F value of Quade nonparametric analysis of covariates;

d, Z value of Mann-Whitney U test;

e, χ2 value of Chi-squared test.

AOO, age of onset; ASMs, antiseizure medications; BN, Boston Naming Test; HC, healthy controls; IQR, interquartile range; MoCA, Montreal Cognitive Assessment; SAS, Self-Rating Anxiety Scale; SD, standard deviation; SDS, Self-Rating Depression Scale; SGS, secondary generalized seizures; Sib-HS, Siblings of TLE-HS; Sib-NHS, Siblings of TLE-NHS; TLE-HS, patients with temporal lobe epilepsy and hippocampal sclerosis; TLE-NHS, patients with temporal lobe epilepsy and without hippocampal sclerosis; VFC, verbal fluency character test; VFP, verbal fluency Pinyin test.
